# Global minds: caregivers’ perceptions of early childhood development across sociocultural contexts

**DOI:** 10.1136/bmjpo-2026-004520

**Published:** 2026-05-26

**Authors:** Mei Elansary, Yousef Khalifa Aleghfeli, Jocelyn Kuhn, Ka Ya Lee, Sarah Strader, Dana McCoy

**Affiliations:** 1 Department of Pediatrics, Boston Medical Center, Boston University Chobanian & Avedisian School of Medicine, Boston, Massachusetts, USA; 2 Global Migration Institute, Toronto Metropolitan University, Toronto, Ontario, Canada; 3 Department of Pediatrics, Emory University School of Medicine, Atlanta, Georgia, USA; 4 Faculty of Education, The University of Hong Kong, Pokfulam, Hong Kong SAR, China; 5 Two Rabbits, Washington, DC, USA; 6 Harvard Graduate School of Education, Cambridge, Massachusetts, USA

**Keywords:** Child Health, Developing Countries, Low and Middle Income Countries, Caregivers, Child

## Abstract

**Background:**

Global scale-up of early childhood development (ECD) interventions, including parenting programmes, has raised concerns about lack of alignment with local sociocultural values and norms. This study explored caregivers’ perceptions of ECD and their role in supporting their children’s development across sociocultural contexts.

**Methods:**

Semi-structured interviews were conducted with 131 primary caregivers of children 0–36 months old in Brazil (n=21), Ghana (n=30), Guatemala (n=30), Hong Kong (n=30) and Lebanon (n=20). Thematic analysis examined common and unique themes related to caregiver perceptions of child intelligence, timing of early learning, child-directed speech and caregiver roles in promoting child development.

**Results:**

Across all sites, caregivers described communication development as an important indicator of intelligence. Specific skills valued across contexts included cognitive alacrity (Guatemala, Hong Kong and Lebanon), preacademic skills (Ghana, Guatemala, Hong Kong and Lebanon) and independence with self-care activities (Brazil and Ghana). With regard to the timing of when children begin learning, caregivers across sites emphasised the developmental period between 1 year and 3 years for when children learn the most. Caregivers identified a range of ages for when they initiated speech with their children, including in the womb (Brazil and Guatemala) and across the first year of life (Ghana and Lebanon). Although caregivers across sites highlighted their role in teaching children socioemotional skills, there were site-specific differences in caregivers’ perceived roles for encouraging other skill domains (eg, preacademic skills in Brazil, motor skills in Ghana).

**Conclusions:**

Findings demonstrate many similarities in caregiver perceptions of ECD across sites as well as site-specific findings that highlight unique caregiver perspectives. These results suggest that parenting interventions should consider targeting early childhood domains prioritised by caregivers across settings while also incorporating local priorities.

**WHAT IS ALREADY KNOWN ON THIS TOPIC**

**WHAT THIS STUDY ADDS**

- This study explored caregivers’ perceptions of early childhood development and their perceived role in supporting children’s development, finding both common and unique themes across different settings.

**HOW THIS STUDY MIGHT AFFECT RESEARCH, PRACTICE OR POLICY**

- Study findings have important implications for the design and scaling up of parenting interventions. Our results suggest that it is possible for interventions to target aspects of development that are prioritised by caregivers across settings (eg, socioemotional and preacademic skills) while also emphasising the specific competencies that are valued locally.

## Introduction

 Research has highlighted children’s development in the first several years of life as critical in predicting later-life success.^1 2^ To promote early childhood development (ECD) on a global scale, the United Nations Sustainable Development Goals (UNSDG) include ECD in goal 4 with the target of ensuring that all children ‘have access to quality ECD, care, and preprimary education’ by 2030 to promote readiness for primary education.^3^ Despite increased recognition of the importance of ECD, an estimated 250 million children mainly from low- and middle-income countries (LMICs)^4^ face risk factors such as poverty and malnutrition that compromise their developmental outcomes.^5^

To address these inequities, multilateral agencies have advocated for investments in programmes and policies that support ECD, including the *Nurturing Care Framework,* which emphasises children’s access to care across five dimensions: responsive caregiving, early learning, safety and security, nutrition and health.^6^ Current estimates suggest that approximately three quarters of children ages 3–4 years in LMICs lack access to minimally adequate nurturing care, as measured by basic indicators from the UNICEF Multiple Indicator Cluster Surveys.^7^ Responsive caregiving—including warm, sensitive and contingent stimulation from mothers and other caregivers—has been highlighted as a particularly important intervention target.^8 9^ Caregiver engagement in stimulation, play and early learning activities has been associated with children’s improved cognitive and socioemotional development, as measured by studies across a variety of LMICs that together represent diverse cultural and economic contexts.^10^,^13^

Interventions to increase responsive caregiving often aim to support parent-child interactions and parent behaviours, knowledge, attitudes and practices during the first 3 years of life.^14^ Numerous parenting programmes have been implemented in diverse global settings, including the popular Reach Up and Learn^15^ and WHO-UNICEF Care for Child Development (CCD) interventions.^16^ Given evidence of their effectiveness across a variety of contexts,^17^ there has been rapidly growing interest in scaling up and disseminating parenting programmes globally.^1^ As an exemplar, the CCD package, which focuses on promoting caregiver-child play and communication to support ECD, has been implemented in 54 LMIC countries.^18^

However, global scale-up of parenting programmes has raised concern for insufficient attention to local values and norms related to parenting and ECD.^19 20^ Though anthropological and psychological research has long emphasised the importance of culture in shaping child development,^21 22^ these insights have not consistently been applied to interventions. This has led to concerns that existing programmes are biased towards definitions and cultural views of healthy child development arising from predominately Minority World^23^ (eg, high-income, Global North) contexts.^24 25^ For example, Reach Up and CCD were developed based on evidence from Minority World contexts. Although both intervention packages emphasise the importance of localisation, their assumptions and content may not align with caregiving practices in diverse settings.^2024 26^,^28^ Studies have highlighted the need for formative research and adaptation of these programmes to local sociocultural contexts.^29 30^

Supporting these concerns, extant research highlights how parents’ goals for their children’s development differ across cultural contexts.^31 32^ For instance, the concept of socially responsible intelligence is recognised as a core cultural model across many African societies,^33^ where caregivers also prioritise children’s sense of belonging, cooperation, obedience and social responsibility.^34^,^36^ Such competencies are rarely reflected in parenting programmes. Additionally, parenting interventions have been criticised for their focus on a single primary caregiver, often the mother, providing cognitive stimulation, when children in many contexts spend considerable time with siblings and extended family.^37 38^ Substantive anthropological work also describes significant differences in beliefs and practices regarding verbal engagement with infants; caregiver-directed speech to young infants as prioritised in Minority World contexts is discouraged in some agrarian societies.^39 40^ Concerns have also been raised about ECD programmes’ emphasis on play, given differences in beliefs and practices around play and children’s early learning.^41 42^ While parents in Minority World contexts may view play as critical for cognitive development,^41^ parents in Majority World^23^ (eg, low- and middle-income, Global South) contexts may emphasise children’s involvement in productive work as central for learning.^43 44^

These potential misalignments between global ECD interventions and local parental goals may threaten the acceptability and effectiveness of parenting interventions implemented in specific local contexts.^45^ Despite the emphasis on scaling and disseminating parenting interventions globally, research on caregivers’ beliefs about ECD remains limited in two critical ways. First, the majority of qualitative research focuses on single contexts, precluding comparison of shared versus context-specific patterns. Second, limited work has examined how beliefs relevant to ECD intervention practices—such as child-directed speech—vary across Majority World contexts. Cross-cultural research on caregivers’ goals and perceptions of ECD and their own role in supporting child development are limited yet critical for informing the science and practice of child development globally.

The present study aims to address these limitations through investigation of caregivers’ conceptualisations of ECD and their perceived role in supporting their children’s development across multiple contexts. The objectives of the present study were to investigate caregivers’ conceptions of child intelligence, beliefs around timing and form of early learning, perceptions of child-directed speech and perceived role in promoting children’s socioemotional development and other developmental domains in daily life. We examined both the similarities and variations in caregivers’ perceptions across sites. Following the model of *universality with specificity*,^46^ we organise our results into common findings across sites as well as unique themes, where relevant.

Several related theoretical frameworks informed this study’s conceptualisation. Specifically, the process-person-context-time model developed by Bronfenbrenner^47^ adds to his bioecological theory of development^48^ by highlighting the significance of proximal processes, which are ongoing and reciprocal interactions that occur between a child and other individuals within their immediate environments (ie, microsystems). We are also informed by Vélez-Agosto’s adaptation of Bronfenbrenner’s model, termed the cultural microsystems model, which describes the ways in which these proximal exchanges between children and caregivers are informed by both distal (ie, macrosystemic) and proximal (ie, microsystemic) sociocultural forces.^49^ In particular, we draw on this model’s definition of culture not just as a singular large-scale macrosystemic influence tied to a particular sociopolitical boundary, but also as a set of proximal, continually evolving forces (eg, relational, political, historical, socioeconomic)^50^ that shape the process and content of daily routines and practices in families. This model emphasises the transactional nature of human development, where sociocultural forces exist within different settings, including media and ECD programmes, that relate to the individual in mutual defining processes.

## Methods

### Settings

This qualitative study was conducted as part of a broader initiative to develop the Caregiver-Reported Early Development Instruments,^51^ a caregiver-reported ECD instrument for global use. This work uses data collected in five country sites: Brazil, Ghana, Guatemala, Hong Kong (Special Administrative Region of China) and Lebanon. Sites were chosen based on (1) voluntary involvement of local research teams conducting ECD research in each area and (2) the goal of obtaining cross-cultural data from diverse sociocultural, political and economic contexts. Specifically, these sites span multiple world regions, capture LMIC contexts as well as large emerging economies^4^ and reflect different value systems,^52^ governance models, healthcare systems and progress towards UNSDG Goals.^53^

### Participants

Primary caregivers of children aged 0–36 months were recruited in all sites. Local research staff recruited a convenience sub-sample of caregivers engaged in research projects and/or via non-governmental organizations and childcare centres (online supplemental file 1 for site characteristics). Although sampling was convenience-based, research staff were asked to recruit participants who were representative of the local population with regard to socioeconomic status, nationality, ethnicity and religion. For example, in Lebanon, both refugee participants and Lebanese participants were included. Researchers also used a sampling strategy whereby caregivers of children aged 0–36 months were recruited in comparable numbers at each site (eg, approximately 1/3 were caregivers of children 0–12 months, 12–24 months and 25–36 months respectively). Sample sizes were also guided by the literature, suggesting sample sizes between 20 and 40 interviews to reach data saturation for metathemes in cross-cultural research.^54^

### Participant involvement

Participants were not involved in the design, conduct, reporting or dissemination plans of the research.

### Data collection and procedures

The study was reviewed by the Harvard institutional review board (IRB) and determined exempt, as it involved interview procedures with no collection of identifiable information. Caregivers provided verbal or written informed consent, in accordance with local IRB requirements, after a field worker read the consent aloud in the preferred local language and answered questions.

Semi-structured indepth interviews were performed with participants in the local language. Interview guides were translated into local languages by multilingual research staff and back translated to English to assess accuracy. Interview questions were purposefully broad to elicit caregiver perceptions regarding ECD (online supplemental file 2). Interviews were conducted by local staff familiar with the local context, including fluency in the local language and knowledge of local norms. Interviews were completed following administration of a caregiver-reported measure of ECD^51^ and lasted approximately 40 min. Interviews were conducted between July 2016 and February 2017 and were audiotaped, transcribed verbatim, translated into English by local field staff using a meaning-based approach^55 56^ to preserve intended meaning over direct verbatim translation for colloquial and culturally bound expressions. All translations were reviewed by bilingual field staff for accuracy. Deidentified transcripts were used for analysis. The analysis team consulted field staff as needed to clarify interpretations.

### Data analysis

Interview transcripts were analysed using thematic analysis to identify patterns across participants through data familiarisation, coding and development of themes (broader shared meaning across participants).^57^ Initially, three investigators were assigned to independently review four transcripts per site using open coding and memoing to organise content and generate an initial codebook with a detailed description of each code.^58^ A priori topics from the interview guide informed code development, and new codes were added inductively.^59^ Using the initial codebook, team members double-coded an additional four transcripts per site and refined the codebook iteratively, reviewing as a group until consensus was reached that codes captured all salient themes across study sites.^60^ The final codebook was then used to double code all transcripts, including those reviewed to generate the codebook, by two coders. The research team met to review coding every four interviews, resolving differences through consensus and discussion with the first author (ME), and consultation of the entire research team as necessary. Intercoder reliability (Cohen’s k) of 81–89% was established with the final code book (see online supplemental file 3 for study codebook). Following coding, thematic analysis was conducted by the coding team who met to review codes and investigate quotes, identify patterns of meaning (themes), examine the relationship of themes to data elements and develop relationships between themes and patterns in the data through axial-coding.^57^ Site-specific and cross-cultural metathemes^54^ were finalised collaboratively. We used NVivo V.12 for qualitative data management.^61^ We followed the Consolidated Criteria for Reporting Qualitative Research (COREQ) (online supplemental file 4).^62^

### Reflexivity

The research team comprised professionals with diverse backgrounds and experiences in ECD, global health, education, psychology, philosophy and community programmes (see online supplemental file 4 for COREQ^62^ Research Reflexivity Statement).^62^ To minimise assumptions, the team held ongoing discussions about their individual biases and beliefs regarding ECD, engaging in regular critical reflection and discussion of their positionality.^63^

## Results

### Demographics

Demographic characteristics of the study sample are provided in table 1. A total of 131 caregivers were interviewed. The majority were mothers (91%), though fathers, grandmothers and other relatives were also included. Educational attainment of caregivers varied, with nearly half (46%) reporting primary or below primary education. In addition to diversity across sites, there was diversity within sites. For example, participants in Lebanon included caregivers who were Lebanese nationals, Palestinian refugees and Syrian refugees, while participants in Guatemala included caregivers who identified as Indigenous and Ladino (non-indigenous or mixed European and indigenous descent).^64^

**Table 1:** Demographic characteristics of study sample

Country	Brazil (n=21)		Ghana (n=30)		Guatemala (n=30)		Hong Kong (n=30)		Lebanon (n=20)		Full sample (n=131)	
	*n*	*%*	*n*	*%*	*n*	*%*	*n*	*%*	*n*	*%*	*n*	*%*
*Child gender*												
Female	10	48%	16	53%	14	47%	13	43%	8	40%	61	47%
Male	11	52%	14	47%	16	53%	17	57%	12	60%	70	53%
*Child age group*												
0–11 months	6	29%	10	33%	10	33%	10	33%	5	25%	41	31%
12–23 months	8	38%	10	33%	9	30%	9	30%	7	35%	43	33%
24–36 months	7	33%	10	33%	11	37%	11	37%	8	40%	47	36%
*Primary caregiver*												
Mother	17	81%	29	97%	27	90%	26	87%	20	100%	119	91%
Father	0	0%	0	0%	1	3%	3	10%	0	0%	4	3%
Grandmother	4	19%	0	0%	1	3%	1	3%	0	0%	6	5%
Other relative	0	0%	1	3%	1	3%	0	0%	0	0%	2	2%
*Caregiver education*												
Below primary	1	5%	19	63%	1	3%	3	10%	8	40%	32	24%
Primary	8	38%	4	13%	5	17%	4	13%	8	40%	29	22%
Secondary	10	48%	0	0%	4	13%	4	13%	0	0%	18	14%
Post-secondary <4 years	0	0%	1	3%	7	23%	2	7%	1	5%	11	8%
Post-secondary ≥4 years	2	10%	4	13%	13	43%	17	57%	2	10%	38	29%
Unknown	0	0%	2	7%	0	0%	0	0%	1	5%	3	2%

Frequencies do not add up to 100% due to rounding.

### Theme 1: Caregivers’ conceptions of child intelligence

To gain understanding of which developmental domains and skills were relevant to caregivers with regards to child intelligence, caregivers were asked what it means to be a smart child and what related skills young children should gain (table 2).

**Table 2:** Caregivers’ conceptions of child intelligence

Theme	Additional illustrative quotes
*Multisite*	
Communication skills development (all sites)	“Being an intelligent child is a child who learns to speak, explain things.” (Mother in Brazil) “If she knows how to talk like saying things you didn’t believe she can say. Or doing things you thought she could not have done, then you can conclude she is smart.” (a Dagomba mother in Ghana) “Beyond knowing things and all that, to me, the most important thing is communication. My child is intelligent because she is able to communicate.” (a Ladino mother in Guatemala)
Cognitive alacrity (Guatemala, Hong Kong and Lebanon)	“[For age 3] I feel that memory is very important. My son, even before 3, he knew where he had been before. That car is a Toyota. That car looks like yours. He has a great memory. I don’t know if that’s an indicator of intelligence, but it’s really important.” (a Ladino mother in Guatemala) “Fast learner. Good memory. For even smarter kids, they can give convincing points and ideas. The kids nowadays are really smart. Say if we teach them 3–3=0. When asked him how about 100–100 or A–A, he knows it’s 0. He may not know unknowns at this stage, but he can deduce it.” (a Chinese mother in Hong Kong) “He understands the information fast, he says it quickly and he always remember it.” (a Palestinian refugee mother in Lebanon)
Socioemotional skills (Guatemala and Hong Kong)	“[For age 1] he is able to recognize people—when children are able to recognize someone, even if they don’t realize it’s a grandfather or grandmother, their behavior, their openness to this person is quite obvious.” (a Ladino mother in Guatemala) “[For age 1] Take the initiative to greet people. He doesn’t have to depend on the parents to do a particular thing.” (a Chinese mother in Hong Kong)
Preacademic skills (Ghana, Hong Kong and Lebanon)	“If he/she is smart he/she should be able to write the name and the English alphabet and numbers… That should be a child who is smart, but if he/she is not smart he/she doesn’t care about all this.” (a Dagomba mother in Ghana) “He can do puzzles.” (a Ladino mother in Guatemala) “For example, I told him the numbers then I said repeat the numbers after me or show me which color, like the red color is number 1, the green color is number 2 then where is number 1 then he will point on it and then you know that he is smart.” (a Palestinian refugee mother in Lebanon)
Independence (Brazil and Ghana)	“Oh, she does not need to use diapers anymore, she eats by herself, she says whatever she wants, if she wants water, if she wants juice, if she wants food or not. That a 3-year-old child already does.” (a mother in Brazil) “If he can undress himself to relieve himself in a chamber pot or any appropriate place without messing himself, then he is smart.” (a Dagomba mother in Ghana)
Motor skills (Brazil, Ghana and Guatemala)	“A one year-old child already knows how to grab, walk, already begins with their intelligence” (a mother in Brazil with primary education) “If you see a one year-old child and they say she is smart then maybe they are referring to how she is able to walk because children walk under two years whiles others do not do so, the smart ones are able to walk under two years. Sometimes she will try to lift certain things by which people around her can say she is smart.” (a Dagomba mother in Ghana with below-primary education) “Well, walking I suppose. Some children walk at a year. My daughter didn’t. She started walking till about a year and a half. Yes, walking.” (an Indigenous mother in Guatemala with primary education)
*Site-specific*	
Play skills (Brazil)	“She already knows how to stack up things, organize thing by size. I notice those things in children, because I take care of boy of 2 years old, when he was 1 year old, he could already play with a car toy and he could put one by one in a line, he could sort things by color, he could distinguish some things.” (a mother)
Physical health and growth (Ghana)	“If the child is not disturbed by so many short illnesses or the child can also be born [intelligent].” (a Dagomba non-parental caregiver)
Sense of responsibility (Ghana)	“If she can wash dirty bowls, you can ask her to do that, if she can also sweep you can ask her to sweep the compound.” (a Dagomba mother)

#### Multisite findings

Caregivers across all sites described communication skills development as an important indicator of intelligence, for example, “Language is a good reflection of how smart is a child,” (a Chinese mother in Hong Kong) and “He has to know all the words” (a Syrian mother in Lebanon). Hong Kong who identified as Chinese. In Guatemala, Hong Kong and Lebanon, caregivers emphasised competencies related to cognitive alacrity as important indicators of intelligence, including: “good memory” (a Ladino mother in Guatemala), “solve problems” (a Chinese mother in Hong Kong) and “understands the information fast” (a Palestinian refugee mother in Lebanon). In Ghana, Guatemala, Hong Kong and Lebanon, caregivers reported preacademic skill development as a salient indicator of intelligence, including early literacy, for example, “able to write” (Dagomba mother in Ghana) and “likes to read” (a Ladino mother in Guatemala), early numeracy, for example, “count to ten” (a Syrian refugee mother in Lebanon), and knowledge of shapes and colours, for example, “that he learns different things: numbers, letters, colors” (a Ladino mother in Guatemala). In Brazil and Ghana, caregivers emphasised independence, as reflected in self-care abilities, as important indicators of intelligence. One mother in Brazil explained, “She is super independent, takes a bath alone, goes into the bathroom alone, if she is hungry she eats alone, I think she is a very independent child, very clever.” In Guatemala and Hong Kong, caregivers valued socioemotional skill development, including recognising family and greeting others, for example, “smile to familiar people” (a Western mother in Hong Kong). In Brazil, Ghana and Guatemala, caregivers with below secondary education valued motor skill development, such as the ability to walk, for example, “A child is smart is when she starts to walk at an early age like less than a year, then you say the child is smart” (a Dagomba mother in Ghana with primary education).

#### Site-specific findings

In Brazil, caregivers often emphasised play and creativity as indicators of intelligence, as one grandmother explained, “Playing with building bricks… he didn’t know it, now he does by himself.” In Ghana, caregivers described physical health and growth as a reflection of a child who is developing optimally, as noted by one mother, “Growing teeth early makes him [smarter].” Caregivers in Ghana also uniquely placed value on children’s developing sense of responsibility, often citing examples of children completing household chores, as one mother shared, “The [smart] child should be able to wash clothes, wash plates, learn how to build fire.”

### Theme 2: Caregivers’ perceptions of timing and indicators of early learning

To elicit caregiver perceptions of the timing and indicators of learning in early childhood, caregivers were asked when children begin learning and when children learn the most and what skills displayed early learning (table 3).

**Table 3:** Caregivers’ perceptions of the timing and indicators of early learning

Theme	Additional illustrative quotes
*Multisite (timing of early learning*)	
Learning happens the most between 1 year and 3 years old (all sites)	“When she is one to two years, she starts to learn plenty things.” (Dagomba mother of a 3-month-old girl in Ghana) “About one year, one year and half, they are more aware of things.” (Mother of a 3-month-old boy in Brazil) “From the age of 1 because some children talk at this age.” (Palestinian refugee mother of a 23-month-old girl in Lebanon)
Learning begins after 1 year of age (all sites; caregivers with up to secondary-level education)	“[Learning begins] since they are 4 or 5 years old. Because I think it is the age that they are open minded for learning.” (Mother of a 23-month-old girl in Brazil with secondary education) “[Learning begins] Normally, from 1 to 5 years of age, they absorb everything. Everything they learn from 1 to 5, they will use for the rest of their lives. The rest, they will learn bit by bit. But the first 5 years are the most important. Why is this? Because the person is formed in the first 5 years.” (Ladino mother of a 14-month-old boy in Guatemala with secondary education) “[Learning begins] around 1.5 years old to 3 years old. They begin to give feedbacks to others. They know how to speak now. So, they are able to voice out their needs and they start to have more demands on us.” (Chinese father of a 26-month-old boy in Hong Kong with secondary education)
Entry into childcare/ school (Brazil and Lebanon)	“[Learning begins] especially when she’s in daycare. Because I put my daughter since she was 1 year old in day care, and at 1 year she already had learned enough. Even now, almost 3 years old, she already speaks things, she already does things that surprises us a lot.” (Mother of a 35-month-old boy in Brazil with primary education) “[Learning begins and happens the most] In kindergarten. 4 and above. Children at this age understand and absorb more. Because the child from 4 and above understand more than when they're older. For example, I don’t understand and absorb information like my daughter. She’s in grade 1 going to grade 2. I put her in this school, they do activities. When she went to grade 1 she came back smarter than our neighbor’s daughter, who is her age. Because the child from age 4 starts understanding and absorbing and can learn how to read and write.” (Syrian refugee mother of a 30-month-old boy in Lebanon with below-primary education)
*Multisite (indicators of early learning*)	
Early motor skills (Ghana and Hong Kong)	“[Learning begins] The way they learn is when they master sitting, she starts to be learning little by little until it starts crawling. The learning continues until also when she then begins to walk, and it continues.” (Dagomba mother of a 10-month-old girl in Ghana) “[Learning happens the most] I think around 1.5 years old, or 1 years old onwards. Because they suddenly understand what you say, they can walk and explore other things. It seems that they learn much more things.” (Chinese mother of a 23-month-old boy in Hong Kong)
Exploration and curiosity (Guatemala and Hong Kong)	“[Learning happens the most at] Two years old. They start to master some ability. They can explore by themselves and from their daily experiences. Through interacting with the environment, they can learn more quickly.” (Chinese mother of a 14-month-old girl in Hong Kong) “[Learning happens the most] probably 8 months to 3 years old. Because they start to be able to move around and everything are new to them. Their curiosity will be strongest. They are initiated to explore things around them. To them, they are like plain papers. They haven’t formed their own judgements and thinking at this moment, so they will absorb the most at this stage.” (Chinese mother of a 7-month-old girl in Hong Kong)
Play (Ghana, Hong Kong and Lebanon)	“[Learning begins] My daughter [mother of child] said ‘play. Group activities.’ Age 1. … [Learning happens the most] Around 2 years old. They learn to talk. Learn to play. They put more focus on play. In the old days, we just played randomly. But now there are more variety of play. Play with cars. Playing himself.” (Chinese grandmother of a 36-month-old boy in Hong Kong with primary education). “[Learning happens the most] from the age of 3 the child likes to play. (why?) because his mind is focusing only on playing and it is the important thing then how to interact with the child and his way of interacting” (Palestinian refugee mother of a 36-month-old girl in Lebanon with primary education).
*Site-specific (timing of early learning*)	
Learning begins in the womb (Guatemala)	“[Learning begins] I think since they are in the mother’s womb. This is where they begin to make out voices. They learn to move since they are little… always.” (Ladino mother of a 21-month-old boy)
Learning begins at birth (Hong Kong)	“Since birth. From the moment she leaves my body or opens their eyes.” (Chinese mother of a 31-month-old girl)
Learning happens most after 3 years of age (Lebanon)	“[Learning happens the most] at 6 years old since they understand more and concentrate more.” (Syrian refugee mother of an 18-month-old girl).

#### Multisite findings

Caregivers identified a range of ages for when they perceived children to begin learning or learn the most. Caregivers across sites emphasised the developmental period between 1 year and 3 years for when children learn the most, for example, “Her learning speed from 1 to 2 years old is a lot faster than 0–1 years old” (Chinese mother of a 31-month-old girl in Hong Kong), “Between 1 and 2, I think their development is much more pronounced” (Ladino mother of a 24-month-old girl in Guatemala) and “[Learning begins and happens the most] since 1 year old. Because when they are babies, I see with my children, they do not have much sense of what they are doing, they imitate what we do. When they are 1 year old, they start showing their own personality. They are learning what they really want” (mother of an 8-month-old boy in Brazil). Caregivers across sites with secondary education and below also often described learning as beginning after age 1 year, for example, “They first start learning when they are 1 year old, since they start to understand. It’s best age to start learning” (Syrian refugee mother of a 12-month-old boy in Lebanon with below-primary education) and “When he is around one and half years or two years then he start learning” (mother of a 20-month-old boy in Ghana with below-primary education). In both Brazil and Lebanon, caregivers with primary and below-primary education often emphasised entry into childcare or school as a marker of learning, as illustrated by a Syrian refugee mother in Lebanon of a 9-month-old boy with primary education, “[Learning begins]… from age 5 and above. In Syria the child goes to kindergarten at this age and then he goes to the first grade… [Learning happens the most when] the child comprehends from a young age; I mean at kindergarten or the first grade they have to start understanding and comprehending. Their minds are empty till they’re like 3 years old.”

Caregivers also described different skills and behaviours when explaining what they perceived to be indicators of when children begin learning and learn the most. In Ghana and Hong Kong, caregivers identified various early motor skills as indicators of learning, for example, “when the child starts to learn how to sit” (mother of a 28-month-old girl in Ghana), “the first time to roll” (Chinese mother of a 17-month-old girl in Hong Kong), “when he starts to crawl” (mother of a 19-month-old boy in Ghana), and “the most important thing is walk” (Chinese mother of a 7-month-old girl in Hong Kong). Caregivers in Guatemala and Hong Kong additionally described children’s curiosity-driven behaviours and exploration of their surrounding environment as indicators of learning, with one Ladino mother of a 35-month-old boy in Guatemala explaining, “When they reach about 24 months, they are more curious to experiment, see and do.” In Ghana, Hong Kong and Lebanon, caregivers with primary and below-primary education often highlighted play as important for learning. For example, a Dagomba mother in Ghana with below-primary education talking about her 33-month-old daughter described learning beginning as, ‘When the child is four to five months old. You can give objects to her to start playing with them. With this she can start learning.”

#### Site-specific findings

In Guatemala, caregivers with postsecondary education and above uniquely expressed that children begin learning in the womb. An indigenous grandmother with postsecondary education interviewed about her 29-month-old granddaughter noted:

“They begin learning from the womb. They begin learning from the moment you find out you are pregnant. If you beginning to stimulate the baby at this point, they will develop normally. They won’t have any delays. If you wait until they are 1 or 2 years old in order for them to start learning, you have wasted all this time.”

In Hong Kong, caregivers often described how children begin learning at birth as described by a Chinese mother of a 14-month-old girl in Hong Kong, “Because since born [birth], the child learns to take in breast milk. The child will keep learning the basic skills to solve her hunger, how to improve her sucking skills, as she grows older.” In Lebanon, some refugee caregivers emphasised that learning happens the most after age 3 years. A Syrian refugee mother of a 12-month-old boy explained, “[Learning happens the most] From 3 years and above because their mind is empty and they can capture everything. They’re very smart at this age. The ages 3–4, they’re very smart.”

### Theme 3: Caregivers’ perceptions of timing and importance of initiating speech with children

Caregivers were asked about their practices and values around initiating speech with children, including when they started speaking to their children (eg, in the womb, at birth, or after birth and within the first 3 months of life) and perceived importance of early communication between caregivers and children (table 4).

**Table 4:** Caregivers’ perceptions of the timing and importance of initiating speech with children

Theme	Additional illustrative quotes
*Multisite (timing of initiating speech*)	
In the womb (Brazil and Guatemala)	“I’ve always talked to my kids since I was preggo. But it’s our moment when I am with my children by ourselves.” (Mother of a 8-month-old boy in Brazil) “I started talking to him as soon as I found out I was pregnant. As he grew and I felt his movements, I asked him why he was moving so much. Things like that.” (Ladino mother of a 24-month-old boy in Guatemala)
Within first 3 months (All sites; caregivers with higher education)	“Because they already listen to us. Since I knew that I was pregnant I already started talking to her.” (Mother of a 35-month-old boy in Brazil with post-secondary education of at least 4 years) “The time I gave birth to him that I started talking to him. I will be holding him and saying your mother has come; your mother has come and dancing with him.” (Dagomba mother of a 34-month-old girl in Ghana with post-secondary education of at least 4 years) “From day 1. Because it’s the main way that humans communicate, using language. It’s a means of communication.” (Ladino mother of a 3-month-old boy in Guatemala with post-secondary education of at least 4 years)
After the first year of life (Brazil, Ghana, Guatemala and Lebanon)	“[at what age do you start to communicate with her?] When she starts to speak let’s say after a year and 2 months, that time she will acquire speech.” (Dagomba mother of a 34-month-old girl in Ghana) “At about a year and a half. That’s why I say that he was a bit lazy. We guessed everything he wanted.” (Ladino mother of a 24-month-old boy in Guatemala)
*Multisite (importance of early communication*)	
For language acquisition (all sites)	“For the development of them. If we stay silent all day long, he will not say anything, he will not know what water is, nothing, so we have to talk.” (Mother of a 23-month-old girl in Brazil) “You have to be speaking to him bit by bit so that he will learn how to speak too. As you speak every day to him, he learns from it.” (Dagomba mother of a 3-month-old boy in Ghana) “It stimulates their understanding at all levels. The more you speak to a baby, the faster he will learn the meaning of words, objects, people’s names, names of objects,” (Ladino mother of a 3-month-old boy in Guatemala)
For establishing a nurturing relationship (all sites)	“If you don’t want both you the child to sit quietly you can be talking to her or playing with her so that all of you don’t feel bored” (Dagomba mother of a 31-month-old girl in Ghana). “The more he feels familiar with your voice, the more secure he feels. He feels comfy when listening to your voice. I think not just me, I expect daddy, older brother and grandma to talk with him too.” (Chinese mother of a 5-month-old boy in Hong Kong) “For me, emotional health is very important. Also, the more you talk to a child the sooner and more quickly he will start talking.” (Lebanese national mother of a 13-month-old boy in Lebanon)
*Site-specific (timing of initiating speech*)	
Caregivers started speaking with child at birth (Hong Kong)	“Since birth. The first few days. ‘ung goo goo’ ‘mama’. I think she wants to hear my voice. Let her know there is somebody who attends to her. Gives her a sense of security.” (Chinese mother of a 7-month-old girl)
*Site-specific (importance of early communication*)	
For child responding to instructions (Ghana)	“My reason is that if we are alone at home he can run some errands for me, such as, fetch water for me, go and bring this, etc, then I’ll tell him ‘well done’. That’s all, sometimes I can tell him to bring rice and we eat… or he should bring yam” (Dagomba mother of a 17-month-old boy)

#### Multisite findings

Caregivers identified a range of ages for when they initiated speech with their children. Caregivers in Brazil and Guatemala emphasised speaking with their child in the womb, for example, “I talked with him from when he was in my stomach” (mother of an 18-month-old boy in Brazil) and “from the womb so that he would recognize my voice” (Ladino mother of a 9-month-old boy in Guatemala). Caregivers in Ghana and Lebanon reported varied responses regarding when they started speaking to their child, ranging across the first year of life, for example, “I start to play and talk with him around 6 to 7 months” (mother of a 17-month-old boy in Ghana) and “Approximately, after three to four months I started talking to him all the time” (Syrian refugee mother of a 10-month-old boy in Lebanon).

Notably, some caregivers in Brazil, Ghana, Guatemala and Lebanon also reported initiating speech after the first year of life, for example, “I started at one year, a year and a half” (mother of a 13-month-old girl in Brazil), “from about one year four month” (mother of an 18-month-old boy in Ghana), “when she turned 2” (Indigenous father of a less than 36-month-old girl in Guatemala) and “from 1 year and half” (Syrian refugee mother of a 36-month-old boy in Lebanon). Of note, caregivers with postsecondary education across all sites in our sample often reported initiating speech within the first 3 months, for example, “I followed programs that said that it’s really important to talk to your child when he’s in the womb” (Lebanese national mother of a 13-month-old boy in Lebanon with postsecondary education) and “Since birth. Talking to her is important. Because I want to talk with her. I love her” (Chinese mother of a 23-month-old girl in Hong Kong with postsecondary education).

Caregivers also provided varied reasons for why they perceived early communication with their child to be important. Caregivers across sites emphasised the importance of early communication for language development, for example, “It stimulates her speech development. More parent-child communication may help facilitate her to share her personal stuff to parents in the future” (Chinese mother in Hong Kong) and “Talking is important with the child to teach him the technical terms that he would face in his daily life” (Palestinian refugee mother in Lebanon). Caregivers across sites also emphasised the importance of early communication for establishing a nurturing relationship, for example, “I want my children to have a friend for any time in their life, for any moment, so I always talk to my children” (Mother in Brazil) and “I think it’s important for them to get to know you … So that he would recognize my voice. It’s the same with his dad; so that he would recognize each other’s voices” (Indigenous mother in Guatemala).

#### Site-specific findings

In Ghana, caregivers emphasised that early communication is important for children to learn to respond to instructions. Explaining why early communication is important, a Dagomba mother in Ghana noted, “Just for simple instructions of collecting things for me. When you want something near, you can let her do it.” In Hong Kong, caregivers emphasised speaking with their child from birth. For example, a Chinese father of a 3-month boy in Hong Kong stated, “Since birth. It’s a linkage—children grow from your voice and your conversation. If you do not give them things, they will not grow. What I meant linkage is relationship and learning.”

### Theme 4: Caregivers' perceived roles in children’s early socioemotional development and other domains

Throughout the interviews, caregivers discussed their perceived role in promoting their children’s learning by highlighting specific caregiving roles related to children’s early development across domains, with particular emphasis placed by respondents on socioemotional learning (table 5).

**Table 5:** Caregivers’ perceived roles in children’s early socioemotional learning and other domains

Theme	Additional illustrative quotes
*Multisite*	
Promoting socioemotional skills (all sites)	“You have to let the child know that if someone like an elder is coming with a load he can go and collect the person. And then go and fetch water for the person to wash his or her legs and then greet the person.” (a Dagomba mother in Ghana) “Empathy. I think this is the most important. Be caring and respectful. Of course, carers have to teach. But the child can observe. Like, oh, that makes other kids unhappy, when he is unhappy, mother and seniors may explore the reasons with him—if it was because of people doing something to me. You have to explain and analyze why he feels such emotions. Let him understand himself more. That applies to analyzing other kids’ emotions too. Like why they would get bad-tempered.” (a Chinese mother in Hong Kong)
Promoting communication (all sites)	“The way he will learn how to speak is for me to talk to him. For instance, if you say something and he also tries to say it. He can say ‘ba’ [meaning father] or when you called him, he responds. He will respond ‘Naab’ [meaning yes].” (a Dagomba mother in Ghana) “Say: ‘thank you’, ‘please’, ‘I'm sorry’, ‘excuse me”; being able to ask for things and being thankful. This is basic. Greeting: Good morning, good afternoon, good night. These are basic things for me.” (a Ladino mother in Guatemala) “When the mother talks to him when he’s a child, like 1 year old, he would repeat what you say like ‘baba’ and ‘mama’.” (a Syrian refugee mother in Lebanon)
*Site-specific*	
Promoting pre-academic skills (Brazil)	“With the ball, her toys. I teach her to see the colors, I show her one color, then I show her another one.” (a Grandmother)
Promoting motor skills (Ghana)	“The child learns new things through the mother, because you will teach the child how to sit, but as for crawling you will not teach him. Every day, I will teach how to sit before I bathe him and feed him before I sit him down again.” (a Dagomba mother)
Teaching responsibility through household chores (Ghana)	“I teach her domestic chores and the older siblings are also teaching her academically.” (a Dagomba mother)

#### Multisite findings

Caregivers across all sites highlighted the importance of caregivers teaching their children socioemotional skills. Examples include caregivers teaching children to demonstrate politeness, for example, “For treating others, he has to be polite and be patient…Parents being an example” (a mother in Hong Kong) and respect for elders, for example, “When he is passing and elders are seated he should squat and greet them or when he wakes up in the morning should go to the father and squat and greet him: good morning” (Dagomba mother in Ghana), modelling appropriate behaviour for children through religion, for example, “I give them the principles that are important for my children: to not be racist or extremist. So I’m taking things from our religion like love and agreement” (Lebanese national mother in Lebanon) and “Parents give education. They teach church stuff. They teach what is wrong, what is right, what you need to do” (a mother in Brazil). Caregivers described teaching children to share with peers, for example, “She needs to know how to share and not be selfish.…When we go outside, we show her that all children are just like her. We don’t tell her she is the only one; we tell her that there are others” (an Indigenous grandmother in Guatemala).

Caregivers across all sites also described how they promote children’s communication skills, for example, “I always talk to my daughter, even if she doesn’t understand it … I think it’s important we talk to her since she’s a baby, not saying wrong words, speaking correctly, so she can learn correctly” (a mother in Brazil) and “You have to communicate with your children. You need to talk with them. It also helps them to express their needs and emotions.” (a Chinese father in Hong Kong).

#### Site-specific findings

Caregivers in Brazil frequently discussed their role in promoting preacademic skills, such as alphabets, numbers, shapes and colours, using play-based activities, as illustrated by a mother in Brazil, “Let’s say, I want him to learn mathematics, so if I make hopscotch from 0 to 10 and I teach him to play, it is the same thing of him learning to count to 10.” Caregivers in Ghana discussed their role teaching children motor skills, in particular the importance of helping a child learn to sit upright, as exemplified by a Dagomba mother in Ghana who stated, “It is the mother who will have to assist her to learn how to sit. As you can see I’m helping her to sit by learning new thing.” Caregivers in Ghana also described promoting responsibility by teaching children to contribute to household chores. For example, when asked about the characteristics a child should possess and who teaches them those skills, a Dagomba mother in Ghana stated, “A child should do what she has been doing for you for others too. When day breaks she wash dishes or laundry for you; she should do same for others too. [Who teaches them?] The parents teach them.”

## Discussion

This study provides valuable insights into caregivers’ perceptions of ECD and their role in shaping development across different sociocultural contexts. We investigated caregivers’ conceptions of child intelligence, beliefs around timing and form of early learning, perceptions of child-directed speech and perceived role in promoting children’s socioemotional development and early learning among caregivers in Brazil, Ghana, Guatemala, Hong Kong and Lebanon. Several key findings emerged. First, caregivers across all sites described children’s communication skills and pre-academic skills (eg, early literacy, numeracy, knowledge of shapes/colours) as important indicators of intelligence. Second, although caregivers identified a range of ages for when they perceive children to begin learning, caregivers across all sites emphasised the developmental period between 1 year and 3 years as when children learn the most. Notably, caregivers across sites with secondary education and below also often described learning as beginning after age 1 year. Third, caregivers identified a range of ages for when they initiated speech with their child. Specifically, across all sites caregivers with higher education reported initiating speech within the first 3 months, whereas some caregivers in Brazil, Ghana, Guatemala and Lebanon reported initiating speech with their children after the first year of life. Despite variations in reported timing, caregivers across all sites described the importance of early child-directed communication not only for acquisition of language skills, but also for establishing a nurturing relationship. Lastly, caregivers articulated specific caregiver roles for children’s skill development, with both shared priorities across sites and site-specific findings. Across all sites, caregivers emphasised their role in teaching socioemotional skills, such as demonstrating politeness and respecting elders, as well as promoting communication skills. Site-specific findings include caregivers in Brazil describing their role in promoting preacademic skills and caregivers in Ghana emphasising their role in helping children develop early motor skills.

Collectively, these findings advance the developmental theory of universality with specificity.^46^ In particular, our study highlights both the ways that early childhood skills may be similarly valued by caregivers across settings (eg, socioemotional and preacademic skills) *and* context-specific priorities (eg, teaching motor skills), supporting earlier work.^31 32^ For example, a key finding from our study is that caregivers across sites valued children’s development of social-emotional skills and their own role in supporting these skills. However, caregivers’ specific priorities for socioemotional development varied across sites, consistent with prior research.^65^,^67^ Caregivers in Ghana emphasised interpersonal skills including politeness and greeting elders. In Guatemala, caregivers highlighted the importance of teaching children to share with others. Caregivers in Lebanon discussed socioemotional skills in relation to religious values and the sociopolitical context.

These findings related to universality with specificity also have important implications for the design and scaling up of parenting interventions. Namely, our results suggest that it is possible for ECD interventions to target aspects of development that are prioritised by caregivers across settings (eg, socioemotional and preacademic skills) while also emphasising the specific competencies that are valued locally. To achieve this balance, our site-specific findings suggest that existing parenting interventions—which, as noted above, are largely based on Minority World frameworks^24 25^—may need to be tailored or supplemented to reflect local caregiver values. For example, parenting interventions in Brazil could incorporate strategies to enhance children’s play and creativity, while programmes in Ghana could emphasise caregiving practices that promote responsibility.

While this study suggests how qualitative research with caregivers may inform locally resonant interventions, findings also demonstrate opportunities to provide education around best practices for intervention for which there is more consensus. For example, the finding that many caregivers—particularly those with lower levels of formal education—report that learning begins after the first year of life highlights an opportunity to introduce evidence around the need to start stimulation and responsiveness from birth. Similarly, in contexts where caregivers report delaying initiation of speech until after the first year of life, leveraging the perspective that early child-directed communication is important for establishing a nurturing parent-child relationship may serve as a salient reinforcer for early communication practices.

The study has several strengths that add to its contribution to the field. First, the study used a rigorous qualitative methodology, including double-coding. Second, the study was conducted in five different sociocultural contexts and included caregivers from LMIC contexts, providing valuable insights into the perceptions of caregivers who have been underrepresented in ECD research. These strengths contribute to the broader literature on ECD and have implications for policymakers, researchers and practitioners working to support ECD in LMIC contexts. The study also has several limitations. First, though consistent with research on best practices in cross-cultural research,^54^ sample sizes for each site were relatively small. The use of convenience sampling to identify participants further limits generalisability (eg, by excluding certain groups of caregivers with different perceptions of ECD), while also complicating interpretation of findings. Namely, we are unable to confirm whether cross-site similarities or differences reflect broader sociocultural forces (eg, cultural values, national policies) versus the influence of the agencies used to recruit participants. It could be, for example, that caregiver perceptions that we interpret as ‘universal’ are actually not shared by other community members who have not participated in ECD programming. In addition, while caregivers’ educational status was collected, we did not assess other sociodemographic factors that may impact caregivers’ perceptions of ECD. Finally, study participants were primarily mothers and future research could benefit from studies that focus on the perspectives of fathers, legal guardians and other extended family members. Future research may adopt ethnographic approaches or expand qualitative investigations to other LMIC contexts and subgroups.

## Conclusions

These findings advance the developmental theory of universality with specificity by demonstrating both shared and context-specific caregiver priorities for ECD. Our preliminary qualitative findings suggest that parenting interventions should target early childhood domains prioritised by caregivers across settings, while also identifying and centering locally defined caregiving priorities that are often absent from standardised intervention curricula. Additional qualitative and quantitative research—particularly in under-represented settings—is needed to better understand localised conceptualisations of ECD and to inform the design and scale-up of parenting interventions that integrate both shared and context-specific caregiving priorities.

## Supplementary material

10.1136/bmjpo-2026-004520 online supplemental file 1

## Data Availability

Data are available upon reasonable request.
