# Supplementary material for: Global minds: caregivers’ perceptions of early childhood development across sociocultural contexts
Source: BMJ Paediatr Open. 2026 May 26;10(1):e004520. doi: 10.1136/bmjpo-2026-004520 (PMC13218195; doi:10.1136/bmjpo-2026-004520)
Supplement: online supplemental file 1 [file bmjpo-10-1-s001.docx]

**Online Supplemental File 1. Site Characteristics of Study Sample**

| **Site** | **Sample Description** | **Language of Interview** | **Ethnic/National Group** |
| --- | --- | --- | --- |
| Lebanon (Tripoli, Bekaa, Beirut, Saida, & Tyre) | Lebanese and refugee (Syrian and Palestinian) children from socioeconomically diverse, urban and rural settings recruited from NGOs and local private nurseries. | Arabic  English | Lebanese (n = 3)  Palestinian (n = 5)  Syrian (n = 12) |
| Ghana (Northern Region) | Representative random sample of six districts in Northern Ghana. All families were enrolled into a behavior change study. Primary source of income is farming; most households below international poverty line. | Dagbani  English | Dagomba (n = 30) |
| Guatemala (Guatemala City, Atitlán, Chimaltenango) | Socioeconomically diverse urban and peri-urban children recruited through a parenting intervention. | Spanish | Indigenous (n = 4)  Ladino (n = 25)  Other (n = 1) |
| Hong Kong SAR, China | Diverse children from urban areas raised by Hong Kong Chinese, Mainland Chinese, and mixed race (often British-/Australian- and Hong Kong Chinese) families. Caregivers are mostly highly educated and middle- and higher-income, recruited through private kindergartens or an NGO. A few caregivers were low-income migrant parents. | Cantonese  Mandarin  English | Chinese (n = 29)  Western (n = 1) |
| Brazil (Sao Paulo) | Representative urban sample of mothers enrolled in public health insurance. Children enrolled in larger cohort study. | Portuguese | Brazilian (n = 21) |

**Online Supplemental File 2. Sample Study Interview Questions**

| **Questions** |
| --- |
| *What does it mean to be a good child? What are important values your child should learn to be a member of your community? How are these values learned?* |
| *When do children first start learning? Is there an age when children learn the most? Why?* |
| *How do children learn new things? What activities do you do in your daily life to help your child learn?* |
| *Is it important to talk to children? Why or why not? When did you start talking to your child? Why? (If not mentioned): How does your child learn language?* |
| *What does it mean to be a smart child? What does a smart child know how to do by age 1? By age 3?* |

**Online Supplemental File 3. Study Codebook**

| **Parent Code** | **Child Node** | **Definition** |
| --- | --- | --- |
| AC: Activity Content | BK - Exploration | Caregiver views books as allowing for their child to explore new concepts and ideas, such as learning about new places, exploring emotions, and discovering concepts. |
|  | BK - Knowledge | Caregiver views books as allowing for their child to learn specific facts and information, such as the alphabet, vocabulary (such as animals, shapes, colors), etc. |
|  | BK - Language | Caregiver views books as allowing the child to learn language and literacy skills, including improving their vocabulary, grammar, intonation, sentence structures, and/or comprehension |
|  | BK - Habit | Caregiver views books as helping the child develop a regular routine of reading and/or an inclination or interest in reading. |
|  | AC - Draw | Child engages in drawing or coloring. |
|  | AC - Adult Communication | The adult talks or gestures with the child, and/or the child talks or gestures with adult(s). |
|  | AC - Peer Communication | The child talks or gestures with other children or youth |
|  | AC - Music | Child listens to or makes music through instruments, clapping, singing, dancing, or other explicit mention of music |
|  | AC - Observe | Child learns by watching and listening surrounding people and things, taking in their behavior. |
|  | AC - Explore | Child learns by exploring new things outdoors (e.g., zoo) |
|  | AC - Physical | Child engages in physical activities such as jumping, playing tags, etc. |
|  | AC - Peer Play | Child engages in joint activities with their siblings or other children of their age, etc. |
|  | AC - Homemade Toys | Caregiver says that the child plays with objects that are made from recycled or unpurchased materials. |
|  | AC - Store-bought Toys | Caregiver says that the child plays with toys that were purchased or manufactured outside home. |
|  | AC - TV | Child watches videos on TV or other platforms |
|  | AC - other | The existing codes don't capture this response. |
|  | AC - skip/ don't know | Caregiver requests to skip the question or replies to the interviewer only by saying, "I don't know." |
| AP: Activity Purpose | AP - Learning | Activity's purpose is educational - for academic, physical/motor, or social learning. |
|  | AP - Play | Activity's purpose is for amusement or enjoyment. |
|  | AP - Help | Activity serves to contribute to family or community needs. |
| CL: Caregiver Learning | CL - SEL | Caregiver believes they play a role in, or explicitly teaches, child social or emotional skills, such as responsibility, morals, or being a good member of the community. |
|  | CL - Academics | Caregiver believes they play a role in, or explicitly teaches, child academic skills such as reading, counting, and other discrete academic knowledge. |
|  | CL - Physical | Caregiver believes they play a role in, or explicitly teaches, child to learn physical skills, such as walking and sitting up. |
|  | CL - Communication | Caregiver believes they play a role in, or explicitly teaches, child how to express self and understand language of others. |
|  | CL - Other | Caregiver believes they play a role in, or explicitly teaches, child other skill not covered by categories 1-4. |
| CT: Relative roles of teachers and parents | CT - Teacher importance | Caregiver says that teachers play more significant roles than parents in supporting the child's general development (domain of development isn't specified). |
|  | CT - Caregiver importance | Caregiver says that parents are more important than teachers in supporting learning and socioemotional well-being of the child |
|  | CT - Caregiver-teacher team | Caregiver says that parents and teachers play equally significant or complementary roles in supprting the child's general development (domain of development isn't specified). |
|  | CT - Caregiver SEL | Caregiver says that parents play a more significant role than teachers in supporting the child's emotional well-being, including relationship skills, self-management, self-awareness, social awareness, and responsible decision-making |
|  | CT - Teacher SEL | Caregiver says that teachers play a more significant role than teachers in supporting the child's emotional well-being, including relationship skills, self-management, self-awareness, social awareness, and responsible decision-making |
|  | CT - Caregiver Learning | Caregiver says that parents play a more significant role than teachers in supporting the child's cognitive and academic development or skill acquisition |
|  | CT - Teacher learning | Caregiver says that teachers play a more significant role than parents in supporting the child's cognitive and academic development or skill acquisition |
|  | CT - other | The existing codes don't capture this response. |
|  | CT - skip/ don't know | Caregiver requests to skip the question or replies to the interviewer only by saying, "I don't know." |
| LB: When learning begins | LB - Womb | Caregiver believes that learning begins in the womb. |
|  | LB - 0-3 months | Caregiver believes that learning begins age 0-3 months |
|  | LB - 4-6 months | Caregiver believes that learning begins age 4-6 months |
|  | LB - 7-11 months | Caregiver believes that learning begins age 7-11 months |
|  | LB - 1 year | Caregivers believe learning begins age 12 months and onward. |
|  | LB - Physical milestones | Caregiver bases their perception of when the child begins learning based on the child's acquisition of motor (gross or fine) skills |
|  | LB - Cognitive milestones | Caregiver bases their perception of when the child begins learning based on the child's acquisition of cognitive skills |
|  | LB - other | The existing codes don't capture this response. |
|  | LB - skip/ don't know | Caregiver requests to skip the question or replies to the interviewer only by saying, "I don't know." |
| LM: When children learn most | LM - 0-3 months | Caregiver believes children learn most at age 0-3 months |
|  | LM - 4-6 months | Caregiver believes that children learn most at age 4-6 months |
|  | LM - 7-11 months | Caregiver believes that children learn most at age 7-11 months |
|  | LM - after 1 | Caregiver believes that children learn most between ages 1-3 years |
|  | LM - after 3 | Caregiver believes that children learn most after age 3 |
|  | LM - Physical Milestones | Caregiver bases their perception of when the child learns most based on the child's acquisition of motor (fine or gross) skills |
|  | LM - Cognitive Milestones | Caregiver bases their perception of when the child learns most based on the child's acquisition of cognitive skills. |
|  | LM - other | The existing codes don't capture this response. |
|  | LM - skip/ don't know | Caregiver requests to skip the question or replies to the interviewer only by saying, "I don't know." |
| ST: When parents start talking to children | ST - Womb | Caregiver starts talking to the child while they are in the womb. |
|  | ST - 0-3 months | Caregiver starts talking to the child at age 0-3 months. |
|  | ST - 4-6 months | Caregiver starts talking to the child at age 4-6 months. |
|  | ST - 7-11 months | Caregiver starts talking to the child at age 7-11 months. |
|  | ST - after 1 | Caregiver starts talking to the child after 12 months. |
|  | ST - Milestones | Caregiver starts talking to the child after the child meets certain developmental milestones. |
|  | ST - other | The existing codes don't capture this response. |
|  | ST - skip or don't know | Caregiver requests to skip the question or replies to the interviewer only by saying, "I don't know." |
| CM: Reason for the importance of communication | CM - Build relationships | Caregiver says that talking to children is important because it establishes connections and familiarity between the parents and the child. |
|  | CM - Convey information | Caregiver says that talking to children is important because it allows the caregiver and the child to exchange information (e.g., instructions, messages, what's going on in school, etc.) |
|  | CM - Learn language | Caregiver says that talking to children is important because it enables the child to acquire vocabulary, morphological, syntactical, and practical knowledge about language. |
|  | CM - other | The existing codes don't capture this response. |
|  | CM - skip/ don't know | Caregiver requests to skip the question or replies to the interviewer only by saying, "I don't know." |
| INT: Conceptions of intelligence | INT - Communication skills | Caregiver defines a smart child in terms of their ability to comprehend, speak or gesture, to convey and/or receive meaning and/or to engage in dialogue. |
|  | INT - Discrete knowledge | Caregiver defines a smart child as a child who knows facts, information, and labels. |
|  | INT - Independence | Caregiver believes that the child demonstrates intelligence by doing different activities autonomously with no or minimal adult support |
|  | INT - Learns independently | Caregiver defines a smart child as a child who is able to acquire new information without being given direct instruction. |
|  | INT - Physical intelligence | Caregiver defines a smart child as a child who has achieved certain physical milestones (e.g. as sitting up, walking). |
|  | INT - Responsibility | Caregiver believes that the child demonstrates intelligence by contributing to family work and activities, supporting shared work, or obeying instructions to complete tasks. |
|  | INT - Solves problems | Caregiver defines a smart child as a child who can apply learned skills independently to different contexts (e.g. using a stool to reach something.) |
|  | INT - other | The existing codes don't capture this response. |
|  | INT - skip/ don't know | Caregiver requests to skip the question or replies to the interviewer only by saying, "I don't know." |

**Online Supplemental File 4. Research Team and Reflexivity Statement based on COREQ Guideline**

Developed from:

Tong A, Sainsbury P, Craig J. Consolidated criteria for reporting qualitative research (COREQ): a 32-item checklist for interviews and focus groups. *International Journal for Quality in Health Care*. 2007. Volume 19, Number 6: pp. 349 – 357

| **No. Item** | **Guide questions/description** | **Response/Reported on Page #** |
| --- | --- | --- |
| **Domain 1: Research team and reﬂexivity** |  |  |
| *Personal Characteristics* |  |  |
| 1. Interviewer/facilitator | Which author/s conducted the inter view or focus group? | ME managed the data collection. Interviews were conducted by local research staff familiar with the local context, including fluency in the local language and familiarity with local customs and cultural expectations. The local research staff had prior experience working on studies related to early childhood and received additional training in qualitative interviewing techniques by the study author (ME) either in person during field visits (Ghana) or remotely (Brazil and Guatemala) or in consultation with data collection partners (Lebanon, Hong Kong) who received training materials developed by study author (ME) with ongoing research support and review of interviews (Page 8). |
| 2. Credentials | What were the researcher’s credentials? E.g. PhD, MD | ME is an MD and assistant professor of pediatrics. YKA is a DPhil in education and a research fellow in data science and migration. JK is a PhD in Educational Psychology and assistant professor of pediatrics. KYL is an PhD in Education and research assistant professor of education. SS is an EdM and founding executive director of Two Rabbits, an NGO serving indigenous communities in Africa. DCM is a PhD in Psychology and Intervention and associate professor in human development and urban education advancement. Local research staff were recommended to have a secondary school level of education, to be literate and fluent in the local languages, and to have experience conducting interviews with caregivers. |
| 3. Occupation | What was their occupation at the time of the study? | At the start of the study, ME and JK were postdoctoral fellows at Boston Children's Hospital. YKA and SS were master’s degree students at Harvard Graduate School of Education. KYL was a doctoral candidate at Harvard Graduate School of Education. DCM was an assistant professor of human development. |
| 4. Gender | Was the researcher male or female? | ME, JK, KYL, SS, and DCM are female. YKA identifies as Gender non-conforming. |
| 5. Experience and training | What experience or training did the researcher have? | All researchers had extensive experience in qualitative research methods, including in conducting interviews, thematic analysis, coding techniques, and qualitative data management using Nvivo. In addition, the first author (ME) completed a two-year graduate program in medical anthropology focused on the qualitative methods and the theory and practice of anthropological research. The local research staff had prior experience working on studies related to early childhood and received additional training in qualitative interviewing techniques (materials developed by ME). Training consisted of a review of the aims of the study, review of the semi-structured interviews, qualitative interviewing techniques, and protocols, as well as basic research best practices (e.g., how to ensure participant confidentiality, data quality, etc.). |
| *Relationship with participants* |  |  |
| 6. Relationship established | Was a relationship established prior to study commencement? | Sites were chosen based on the voluntary involvement of local research teams conducting early childhood research in each area. Data collection partners had pre-existing relationships with senior author (DCM) and Caregiver Reporter Early Childhood Development Index Study Team (Page 7). |
| 7. Participant knowledge of the interviewer | What did the participants know about the researcher? e.g. personal goals, reasons for doing the research | Caregivers were informed of the study objectives. The researchers had no personal goals or reasons for doing the research beyond completing the aims and objectives of the study. |
| 8. Interviewer characteristics | What characteristics were reported about the inter viewer/facilitator? e.g. Bias, assumptions, reasons and interests in the research topic | The local interviewers declared themselves and the aims and objective of the research project and reasons for interest in the topic. This was beneficial for the semi-structured interview format as the interviewers were able to probe and ask for clarification where needed. Throughout data analysis, the authors had ongoing discussions about their individual biases and beliefs regarding ECD to minimize their biases and assumptions. They engaged in regular critical reflection and discussion of their own positionality (Page 10). |
