# Supplementary material for: Global minds: caregivers’ perceptions of early childhood development across sociocultural contexts
Source: BMJ Paediatr Open. 2026 May 26;10(1):e004520. doi: 10.1136/bmjpo-2026-004520 (PMC13218195; doi:10.1136/bmjpo-2026-004520)
Supplement: Supplementary file 2 [file bmjpo-2026-004520.reviewer_comments.pdf]

PEER REVIEW HISTORY

BMJ Paediatrics Open publishes all reviews undertaken for accepted manuscripts. Reviewers are asked to complete a checklist review form and are provided with free text boxes to elaborate on their assessment. These free text comments are reproduced below.

ARTICLE DETAILS

**TITLE (PROVISIONAL)**	Global Minds: Caregivers' perceptions of early childhood development across sociocultural contexts
**AUTHORS**	Elansary, Mei<br>Aleghfeli, Yousef Khalifa<br>Kuhn, Jocelyn<br>Lee, Ka Y<br>Strader, Sarah<br>McCoy, Dana

VERSION 1 – REVIEW

**REVIEWER NAME**	Garda, Laila
**REVIEWER AFFILIATION**	KEM Hospital Pune Research Centre
**REVIEWER CONFLICT OF INTEREST**	No Conflict of Interest
**AI DISCLOSURE**	Grammarly for checking language and for any correction required.
**DATE REVIEW RETURNED**	10-Mar-2026

**GENERAL COMMENTS**	Manuscript summary<br>This manuscript examines caregivers' perceptions of early childhood development (ECD) and their role in supporting child development across varied cultural settings. It examines similarities and differences in caregiver beliefs and practices. This has important implications for scaling up caregiver interventions to address ECD. The manuscript is essentially robust and addresses important aspects of a qualitative study, but has a few limitations.<br>Positive points in the study<br>• The paper clearly recognises the knowledge gap it addresses.<br>• It shows a cross-cultural scope involving 5 countries<br>• Usage of Qualitative interviews (semi-structured) permits getting answers in participants' own words<br>• The analysis process is rigorous and well-explained<br>Points that require attention<br>In the introduction, the study's objectives need to be explained.<br>Methodology<br>Why were these 5 countries selected? Though some explanation is given, how these countries contribute to cross-cultural perceptions needs to be defined.<br>There appears to be limited clarity in the recruitment process.<br>In translating interviews into English, cross-cultural significance may be lost, so elaboration on the translation process would add value.<br>Results<br>Some more direct participation quotes would enhance the representation of cross-cultural nuances.<br>Limitations<br>The limitations section needs to be explained in more detail.<br>By using convenience sampling, certain caregiver groups may have been excluded; hence, the effect this may have on the findings

	needs to be addressed.<br>Conclusions<br>These need to be framed more carefully, based on the findings, considering the sample size of 131 and the study's qualitative nature.<br>Overall, the manuscript addresses an important and relevant topic in global early childhood development. With further clarification of methodological details and other sections, the manuscript can contribute positively to the literature.

VERSION 1 – AUTHOR RESPONSE

Reviewer: 1
Dr. Laila Garda, KEM Hospital Pune Research Centre
Comments to the Author
Manuscript summary
This manuscript examines caregivers' perceptions of early childhood development (ECD) and their role in supporting child development across varied cultural settings. It examines similarities and differences in caregiver beliefs and practices. This has important implications for scaling up caregiver interventions to address ECD. The manuscript is essentially robust and addresses important aspects of a qualitative study, but has a few limitations.
Positive points in the study
• The paper clearly recognises the knowledge gap it addresses.
• It shows a cross-cultural scope involving 5 countries
• Usage of Qualitative interviews (semi-structured) permits getting answers in participants' own words
• The analysis process is rigorous and well-explained
Points that require attention

In the introduction, the study's objectives need to be explained.

Response: We thank the reviewer for this suggestion. The text (pgs. 6-7) now clarifies:

The present study aims to address these limitations through investigation of caregivers' conceptualizations of ECD and their perceived role in supporting their children's development across multiple contexts. The objectives of the present study were to investigate caregivers' conceptions of child intelligence, beliefs around timing and form of early learning, perceptions of child-directed speech, and perceived role in promoting children's socio-emotional development and other developmental domains in daily life. We examined both the similarities and variations in caregivers' perceptions across sites. Following the model of *universality with specificity*,[46] we organize our results into common findings across sites as well as unique themes, when relevant.

Methodology
Why were these 5 countries selected? Though some explanation is given, how these countries contribute to cross-cultural perceptions needs to be defined.

Response: We appreciate the reviewer's suggestion. We provide additional rationale for selection of these countries in the methods section.

The methods (pg. 7) now clarify:

Sites were chosen based on 1) voluntary involvement of local research teams conducting ECD research in each area; and 2) the goal of obtaining cross-cultural data from diverse sociocultural, political, and economic contexts. Specifically, these sites span multiple world regions, capture LMIC contexts as well as large emerging economies,[4] and reflect different

value systems, [52] governance models, health care systems, and progress towards UNSDG Goals.[53]

There appears to be limited clarity in the recruitment process.

Response: We regret that more clarity is needed in the recruitment process.

The methods (pg. 8) now provide additional details:

Primary caregivers of children aged 0-36 months were recruited in all sites. Local research staff recruited a convenience sub-sample of caregivers engaged in research projects and/or via NGOs and childcare centers (see online supplemental file 1 for site characteristics). Although sampling was convenience-based, research staff were asked to recruit participants who were representative of the local population with regards to socioeconomic status, nationality, ethnicity, and religion. For example, in Lebanon, both refugee participants and Lebanese participants were included.

In translating interviews into English, cross-cultural significance may be lost, so elaboration on the translation process would add value.

Response: We appreciate the reviewer's concern that cross-cultural meaning can be lost during translation. We have added further details regarding our translation procedures:

The text (pg. 9) now clarifies:

Interviews were conducted between July 2016 and February 2017, and were audiotaped, transcribed verbatim, translated into English by local field staff using a meaning-based approach[55, 56] to preserve intended meaning over direct verbatim translation for colloquial and culturally bound expressions. All translations were reviewed by bilingual field staff for accuracy. De-identified transcripts were used for analysis. The analysis team consulted field staff as needed to clarify interpretations.

Some more direct participation quotes would enhance the representation of cross-cultural nuances.

Response: We agree with the reviewer that additional direct participant quotations will enhance the representation of cross-cultural findings. In response, we have added additional exemplar quotations for findings detailing multi-site findings, ensuring that representative quotes from each setting are represented.

The results (pg. 11) pertaining to caregiver's conceptions of intelligence and the importance of communication skills now include a participant quote from Lebanon:
Caregivers across all sites described communication skills development as an important indicator of intelligence e.g., "Language is a good reflection of how smart is a child," (Chinese mother in Hong Kong) and "He has to know all the words" (Syrian mother in Lebanon). Hong Kong who identified as Chinese. In Guatemala, Hong Kong, and Lebanon, caregivers emphasized competencies related to cognitive alacrity as important indicators of intelligence, including: "good memory" (Ladino mother in Guatemala), "solve problems" (Chinese mother in Hong Kong), and "understands the information fast" (Palestinian refugee mother in Lebanon).

Findings related to pre-academic skills as an indicator of early learning (pg. 12) now includes a quote from a mother in Guatemala:

In Ghana, Guatemala, Hong Kong, and Lebanon, caregivers reported pre-academic skill development as a salient indicator of intelligence, including early literacy, e.g., "able to write" (Dagomba mother in Ghana) and "likes to read" (Ladino mother in Guatemala), early numeracy, e.g., "count to ten" (Syrian refugee mother in Lebanon), and knowledge of shapes

and colors e.g., “that he learns different things: numbers, letters, colors” (Ladino mother in Guatemala).

Additional participant quotes were added to the results related to caregiver’s perceived roles in children’s early socio-emotional learning (pgs. 17-18) as previously described.

Limitations
The limitations section needs to be explained in more detail.
By using convenience sampling, certain caregiver groups may have been excluded; hence, the effect this may have on the findings needs to be addressed.

Response: We regret that sufficient detail was not provided with regards to limitations, We have now expanded the limitations sections to include limitations of convenience sampling.

The discussion (pg. 21) now reads:

The use of convenience sampling to identify participants further limits generalizability (e.g., by excluding certain groups of caregivers with different perceptions of ECD), while also complicating interpretation of findings. Namely, we are unable to confirm whether cross-site similarities or differences reflect broader sociocultural forces (e.g., cultural values, national policies) versus the influence of the agencies used to recruit participants. It could be, for example, that caregiver perceptions that we interpret as “universal” are actually not shared by other community members who have not participated in ECD programming.

Conclusions
These need to be framed more carefully, based on the findings, considering the sample size of 131 and the study's qualitative nature.

Response: We thank the reviewer for this important concern. We agree that the findings should be framed more carefully to reflect the preliminary nature of the findings. We have revised the implications accordingly, noting that these results are preliminary and discuss the need for more qualitative and quantitative research.

The conclusions (pg. 22) now state:

These findings advance the developmental theory of universality with specificity by demonstrating both shared and context-specific caregiver priorities for ECD. Our preliminary qualitative findings suggest that parenting interventions should target early childhood domains prioritized by caregivers across settings, while also identifying and centering locally defined caregiving priorities that are often absent from standardized intervention curricula. Additional qualitative and quantitative research – particularly in underrepresented settings - is needed to better understand localized conceptualizations of ECD and to inform the design and scale-up of parenting interventions that integrate both shared and context-specific caregiving priorities.

Overall, the manuscript addresses an important and relevant topic in global early childhood development. With further clarification of methodological details and other sections, the manuscript can contribute positively to the literature.

Reviewer: 1
If you have selected ‘Yes’ above, please provide details of any competing interests.: Not applicable
